# Biofilm eradication and antifungal mechanism of action against *Candida albicans* of cationic dicephalic surfactants with a labile linker

**DOI:** 10.1038/s41598-021-88244-1

**Published:** 2021-04-26

**Authors:** Emil Paluch, Jakub Szperlik, Łukasz Lamch, Kazimiera A. Wilk, Ewa Obłąk

**Affiliations:** 1 Department of Microbiology, Faculty of Medicine, Wroclaw Medical University, Tytusa Chałubińskiego 4, 50-376 Wrocław, Poland; 2 Department of Genetic Biochemistry, Faculty of Biotechnology, University of Wroclaw, Przybyszewskiego 63, 51-148 Wrocław, Poland; 3 Department of Engineering and Technology of Chemical Processes, Faculty of Chemistry, Wrocław University of Science and Technology, Wybrzeże Wyspiańskiego 27, 50-370 Wrocław, Poland; 4 Department Physicochemistry of Microorganisms, Faculty of Biological Sciences, University of Wrocław, Przybyszewskiego 63/77, 51-148 Wrocław, Poland

**Keywords:** Fungal biology, Biofilms, Antifungal agents, Cellular microbiology

## Abstract

Our research aims to expand the knowledge on relationships between the structure of cationic dicephalic surfactants—*N*,*N*-bis[3,3_-(dimethylamine)propyl]alkylamide dihydrochlorides and *N*,*N*-bis[3,3_-(trimethylammonio)propyl]alkylamide dibromides (alkyl: n-C9H19, n-C11H23, n-C13H27, n-C15H31)—and their antifungal mechanism of action on *Candida albicans*. The mentioned groups of amphiphilic substances are characterized by the presence of a weak, hydrochloride cationic center readily undergoing deprotonation, as well as a stable, strong quaternary ammonium group and alkyl chains capable of strong interactions with fungal cells. Strong fungicidal properties and the role in creation and eradication of biofilm of those compounds were discussed in our earlier works, yet their mechanism of action remained unclear. It was shown that investigated surfactants induce strong oxidative stress and cause increase in cell membrane permeability without compromising its continuity, as indicated by increased potassium ion (K^+^) leakage. Thus experiments carried out on the investigated opportunistic pathogen indicate that the mechanism of action of the researched surfactants is different than in the case of the majority of known surfactants. Results presented in this paper significantly broaden the understanding on multifunctional cationic surfactants and their mechanism of action, as well as suggest their possible future applications as surface coating antiadhesives, fungicides and antibiofilm agents in medicine or industry.

## Introduction

Multifunctional surfactants constitute a group of single molecules comprising several functions, attributed to appropriate hydrophilic or hydrophobic groups as well as linking, branching or counterion moieties. One of the most interesting groups of multifunctional surfactants comprises dicephalic cationic surfactants with an ester or amide linker. The cationic center, most often a quaternary ammonium salt, is not only responsible for water solubility but is also known for its antimicrobial activity. A labile linking group, such as an ester or amide, is responsible for faster biodegradation of the surfactant molecule, meeting the requirements of “green chemistry” regarding newly synthesized amphiphiles. Generally, the lifecycle of surfactants is closely connected with two groups of very hazardous pollutants: by-products and waste from the industrial processes of their synthesis and purification as well as their remnants in wastewater, environment and even living organisms. Some of them are particularly persistent and prone to accumulate in tissues, due to slow and limited biodegradation. In order to prevent contamination, linked to use of surfactants, two general approaches are continuously being developed. One of them involves the use of environmentally friendly raw materials and processes, e.g. renewable oils and fats instead of petrochemicals, as well as solventless synthetic routes. On the other hand, it is strongly preferred to introduce into the surfactant molecule appropriate moieties, enabling its hydrolysis and/or biodegradation, such as a straight, aliphatic tail instead of a branched or aromatic one as well as labile, e.g. ester, amide, carbamate or disulfide bonds, linking groups between hydrophobic and hydrophilic parts^1^.

Dicephalic cationic surfactants with an amide linking group due to their biodegradability do not accumulate in the environment, and in the future may be used as effective antifungal compounds in industry as well as medicine. It will provide for a more environmentally friendly solution than those presently in application. Among the most widespread and efficient disinfecting agents, known also for their biofilm-preventing activity, are compounds containing a quaternary ammonium group, most often coupled with hydrophobic or amphiphilic moieties such as alkyl or polymeric chains^2,3^.

So far a significant number of quaternary ammonium surfactants with custom-designed chemical structures have been prepared: double tail and single head, double head and single tail, gemini type and multimeric amphiphiles^4–6^. The most prominent feature of quaternary ammonium-based surfactants is their significant antibacterial and antifungal activity, which is important in the light of their possible biomedical applications^7–11^.

*Candida albicans* is an opportunistic pathogen occurring as a component of the microbiome of the human digestive tract and reproductive system. Disturbances of function of the human immunological system or composition of the microbiome itself could result in candidiasis^12,13^. These comprise dermal infections and superficial mucosal, such as thrush, vaginal yeast infections to hematogenously disseminated infection with high mortality (approaching 40% in some cases)^14,15^. Such infections are especially dangerous to patients who are infected by HIV, taking immunosuppressive drugs, or have had medical hardware implanted (implants, endoprosthesis, vascular catheter)^16^.

**Figure 1:**
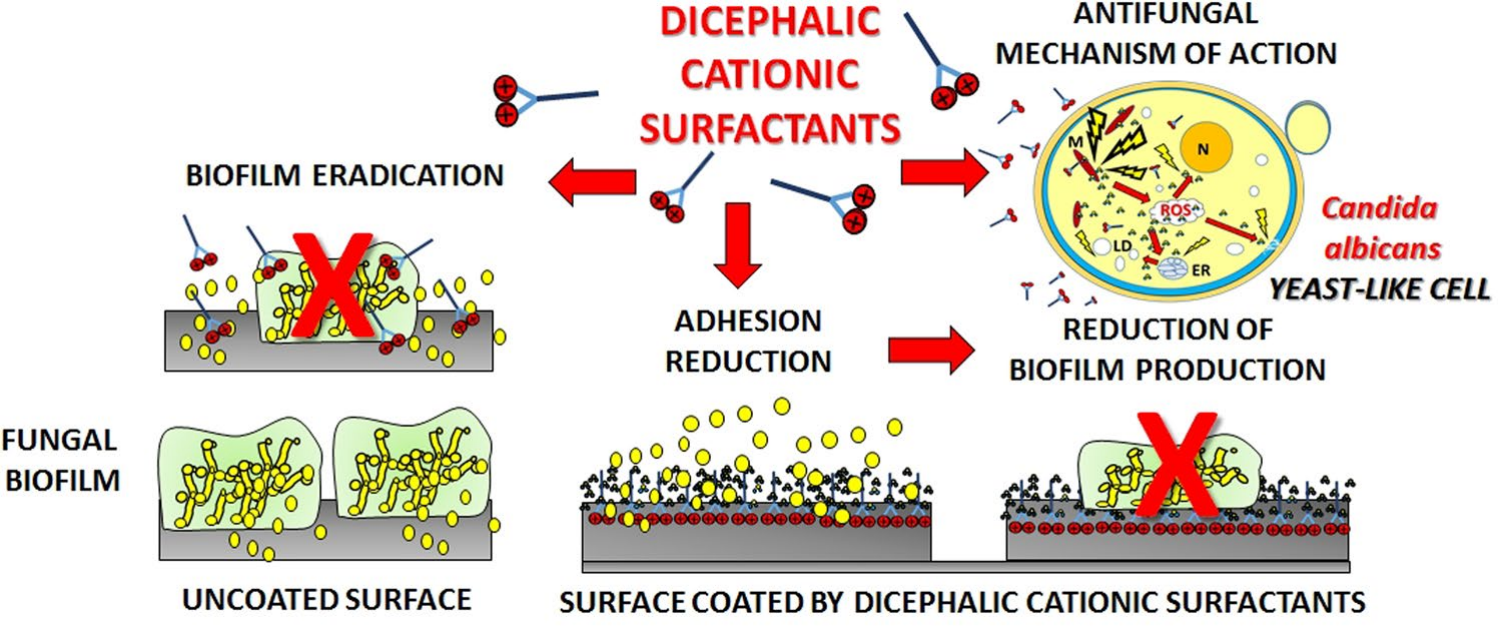
Multifunctional mechanism of action of dicephalic cationic surfactants. Multifunctional mechanism of action of investigated surfactants, taking into account the relation of abiotic surfaces with formation and eradication of *C. albicans* biofilm especially, as well as the structure of yeast cells and the molecular mechanism of action of the surfactants.

Dicephalic cationic surfactants possess a double positive charge which translates to stronger interaction with negatively charged cell structures. On the other hand, their alkyl chains may strongly interact with hydrophobic structures of fungal cells, of which the hydrophobic properties may vary in strength depending on the type and developmental stage of a given cell^17^. These interactions may lead to intercalations of the surfactants into cell membranes and potentially their disruption, alkylation of surface proteins, as well as penetration into the cell and damage to mitochondrial functions and acute oxidative stress (Fig. 1)^18–21^.

As one of the most important stages of biofilm formation is the adhesion of planktonic cells to a surface, so cationic surfactants able to coat a surface and modify its properties should be able to disrupt this process, which is crucial in biofilm formation of for example *Staphyloccocus*, *Pseudomonas* or *Candida*^9,11,18^. It could mimic the action of other compounds known to possess antiadhesive properties, as mannosides competing for adhesion sites with FimH adhesines, or pilicids and curlicids that interact with pili of types I and curly, respectively, which leads to significant reduction in biofilm formation^22^. Utilization of enzymes to degrade extracellular DNA (eDNA) and degrade the matrix of biofilm is also a possible^23^.

In our work we hope to draw attention to the problem of the mechanism of action of investigated surfactants, taking into account the relation of abiotic surfaces with formation and eradication biofilm of *C. albicans* especially, as well as the structure of yeast cells and the molecular mechanism of action of the surfactants.

## Materials and methods

### Surfactants

**Figure 2:**
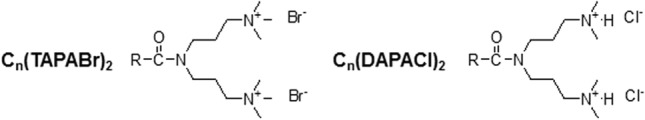
Chemical structures of dicephalic surfactants.

The dicephalic *N*,*N*-bis[3,3′-(dimethylamine)propyl]alkylamide dihydrochlorides (C_n_(DAPACl)_2_) and *N*,*N*-bis[3,3′-(trimethylammonio) propyl]alkylamide dibromides (C_n_(TAPABr)_2_) were synthesized according to procedures described before^2,24^. The greener synthetic routes of the mentioned surfactants’ intermediates^24^ enabled us to use fatty acids as raw materials instead of their chlorides. Moreover, solventless synthetic route, utilizing use of solvent still-head distillation apparatus, made it possible to avoid employment of chlorocarbon solvent and extraction steps, leading to formation of harmful waste. Briefly, tetradecanoic or hexadecanoic acid (54 mmol), 3,3′-iminobis(*N*,*N*-dimethylpropylamine (68 mmol), and NaF (5 mmol) were placed in a reaction vessel, equipped with a solvent still-head distillation apparatus filled with dry Al_2_O_3_, followed by heating at 180–185 °C with continuous dry N_2_ flow for 24 h. After reaction completion, residual 3,3′-iminobis(*N*,*N*-dimethylpropylamine was removed in vacuo and the crude products were dissolved in acetone, filtered and evaporated to dryness under reduced pressure. The obtained semiproducts (40 mmol) were allowed to react with 0.5 m HCl (200 mL) at 5 °C for 5 h and the resulting products C14(DAPACl)2 and C16(DAPACl)2 were isolated by freeze-drying. Yield: 70–75%. For C14(TAPABr)2 and C16(TAPABr)2 the semiproducts (40 mmol), obtained in reaction of appropriate fatty acid with 3,3′-iminobis(*N*,*N*-dimethylpropylamine, were allowed to react with excess of bromomethane solution in anhydrous ethyl ether (400 mL) at 0 °C for 24 h. The precipitated products were filtered, washed with two portions of cold ethyl ether (2 × 25 mL) and dried in vacuo. Yield: 75–80%. The most fungicidally active surfactants were chosen to be investigated: (C_14_(TAPABr)_2_ ,C_16_(TAPABr)_2_, C_14_(DAPACl)_2_, C_16_(DAPACl)_2_), based on previous research^18^. The chemical structures of dicephalic surfactants are shown in Fig. 2. *N*,*N*-bis[3,3′-(dimethylamine)propyl]alkylamide dihydrochlorides (C_n_(DAPACl)_2_ are pH sensitive compounds, due to presence of amine hydrochloride cationic moiety, with tendency to form free amine in basic solutions, resulting in significant drop of aqueous solubility. On the other hand *N*,*N*-bis[3,3′-(trimethylammonio)propyl]alkylamide dibromides (C_n_(TAPABr)_2_ comprise group of relatively pH-stable cationic surfactants with quaternary ammonium salt as hydrophilic group, although undergoing degradation in strongly basic environments. The chemical structures of dicephalic surfactants are shown in Fig. 2 and extended data in Electronic Supplementary Materials (Table 1).

### Strains and growth conditions

*Candida albicans* (ATCC 10231) was used to study the mechanism of action of the double-headed cationic surfactants. *C. albicans* was purchased from the American Type Culture Collection (LGC France SARL, Strasbourg, France). Yeast Peptone Glucose (YPG; 1% Difco Yeast extract, 1% Difco peptone, 2% Difco glucose) was used to cultivate the strains. Obtained cultures were centrifuged, washed with PBS (pH 7.4) and suspended in fresh YPG so suitable optical density was achieved, according to experimenter’s judgment.

### Minimal inhibitory and fungicidal concentration

The values of the minimal inhibitory concentration (MIC) and minimal fungicidal concentration (MFC) were assessed according to published protocols^18^. MFC was expressed as the concentration of the dicephalic surfactant that reduced the number of colony forming units on YPG medium (CFU) by 99.9% after 24 h of incubation at 37 °C^25^. MIC and MFC of tested compounds were also measured for this work, being evaluated by dilution in liquid RMPI 1640 medium in 96 well microplates, using methodology M27-A4 of the CLSI^26^.

### Microscopy

*Candida albicans* ATCC 10231 cultures were centrifuged and diluted in PBS to OD 0.6. Surfactants were added to the cultures to the final concentration of ½ MIC, unless specified otherwise. Untreated cells were used as a control. Carl Zeiss Axio Imager M1 microscope with an AxioCam MRc5 camera was used to visualize the results. Three random fields of view per experimental condition were observed. Acquired images were processed and analyzed in the Fiji/ImageJ software (NIH). The areas from binarized images were then transferred onto original background channel (DIC or Calcofluor white MR2) and for individual fluorescent probes channels MIP images and mean fluorescence intensities of all detected objects per field of view were calculated using the ImageJ's Analyze Particles function (Software Fiji/ImageJ software ver. 1.53c).

#### Oxidative stress

##### General oxidative stress

2′,7′-dichlorofluorescin diacetate (DCFH-DA) was used to evaluate oxidative stress. *C. albicans* cells suspended in PBS 0.6 OD, were incubated for 30 min with 2 μM DCFH-DA (Sigma-Aldrich, USA). Green fluorescence of cells undergoing oxidative stress was observed under microscope (filter 38 HE λex = 495 nm, λem = 517 nm).

##### Production of superoxide anion

The production of superoxide (O_2_·^−^) was evaluated using dihydroethidium (DHE). *C. albicans* cells suspended in PBS 0.6 OD, were incubated for 30 min with 2 μM DHE (Sigma-Aldrich, USA). Yellow fluorescence producing superoxide (O_2_·^−^) were observed under a Carl Zeiss Axio ImagerM1 microscope with 43 HE filter (λex = 460 nm, λem = 640 nm).

##### Mitochondrial oxidative stress

2 μM of Mitosox Red mitochondrial superoxide indicator (MR-MSI) (Sigma-Aldrich, USA) and 4 μM Calcofluor White M2R (Sigma-Aldrich, USA) were used to observe mitochondrial oxidative stress. Yeast cells in PBS 0.6 OD, were incubated with both dyes for 30 min. 49 HE (λex = 370 nm, λem = 420 nm) and 43 HE (λex = 510 nm, λem = 580 nm) filter were used for MR-MSI and Calcofluor White, respectively.

#### Disruption of the cells membrane

Samples were incubated for 30 min with 2 μM Sytox Green Dead Cell Stain (Sigma-Aldrich) and 4 μM Calcofluor White M2R (Sigma-Aldrich). For Calcofluor White M2R dye 49 HE filter was used (λex = 370 nm, λem = 420 nm), while for Sytox Green Dead Cell Stain 38 HE filter was used (λex = 504 nm, λem = 523 nm) to observe red fluorescence of dead cells with disrupted cell’s membrane continuity.

### Transmission electron microscopy (TEM)

*Candida albicans* ATCC 10,231 cells were suspended in PBS and diluted to 1.0 OD. Then resulting suspension was centrifuged for 5 min, at 5 000 rpm. Pellet was consequently incubated for 8 h in 4% glutaric aldehyde pH 7.4, and then washed for 24 h in 0.2 M PBS pH 7.4, finally preserved for 2 h in 2% osmium oxide (VIII). The cells were then centrifuged for 5 min, at 3 000 rpm and washed for 30 min in redistilled water. Clean samples were dried in progressively more concentrated series of 50%, 70%, 80%, 90%, 96%, 100% alcohol-acetone three times, incubated for 15 min in each of the solutions. Dehydrated cells were suspended in acetone:epon 812 mixture (1:1) and incubated in it for 16 h. Then saturated cells were immersed in Epon 812 epoxy and polymerization was carried out for 24 h at 45 °C and 60 °C. Epoxy semi droplets were cut into semi-thin sections and ultrathin sections with a diamond knife (Reichert-Jung, Germany). Then resulting ultrathin sections were treated for 15 min with 2% solutions of uranyl acetate and lead citrate for contrast. Finally prepared samples were observed under transmission electron microscope (TEM) Tesla BS-540^27^. Untreated cells served as a control.

### Leakage of potassium and calcium ions

The leakage of potassium and calcium ions from yeast cells treated with dicephalic surfactants was estimated according to Obłąk et al., 2016^28^. *C. albicans* cultures grown overnight were suspended in 50 mM glucose solution (pH of 6.0) until OD 0.8 was obtained. Then dicephalic cationic surfactants at the concentrations corresponding to MIC were added and samples were incubated for 15 min at 28 °C. The samples were then centrifuged at 4000 rpm for 5 min and the supernatants were analyzed by atomic emission spectrometer (Varian AA240FS). Untreated cells were used as negative control, while autoclaved *C. albicans* cells were used as a positive control.

### Biofilm eradication

*Candida albicans* biofilm eradication on glass surfaces was assessed according to procedure: 3 ml of *C. albicans* culture, 10^6^ CFU/ml in RMPI 1640 MOPS buffered medium, was pipetted into wells of 6 well plates, and then sterile round microscope slides were put in the wells (Ø 15 mm). Cultures were incubated at 37 °C for 24 h with shaking (240 rpm). Subsequently microscopic slides were washed twice with sterile physiological salt solution and transferred to a new 6 well plate, where chosen cationic surfactants were added to final concentrations ranging from 50- to 1000 µM and incubated for 2 h at 37 °C. The slides were washed twice with sterile physiologic salt solution and then transferred to fresh 6 well plate, where the biofilm was stained by 5 min. incubation with 100 μl 2,45 µM of crystal violet. The slides were then washed three times with sterile physiological salt solution. Crystal violet artifacts were dissolved by washing with 1 ml of washing solution (isopropanol, HCl 50 mM, SDS 1%). Absorbance was then read at λ = 590 nm (ASYS UVM 340 Biogenet). Not treated cells were used as control. The experiment was carried out in triplicate.

### Cell viability in biofilm (CLSM)

Aliquots of 3 ml of *C. albicans* ATCC 10,231 in RMPI 1640 MOPS buffered medium 10^6^ CFU/ml were added to the wells of sterile 6 well plates. Sterile microscopic slides (Ø 15 mm) were put in the wells and the resultant cultures were incubated at 37 °C for 24 h with shaking (240 rpm). The slides were washed twice with sterile physiological salt solution and transferred to fresh 6 well plates. *C. albicans* biofilm on glass was treated with chosen surfactants: C_14_(DAPACl)_2_; C_16_(DAPACl)_2_ at 50 and 1000 μM were stained with 3 µl propidium iodide (Ex λ = 543 nm) and 3 µl SYTO 9 (Ex λ = 488) for 3 ml using LIVE/DEAD BacLight Bacterial Viability Kit (Thermo Fisher Scientific). The imaging was performed on an upright Leica SP8 resonant scanning confocal system equipped with spectral PMT detectors (Leica Microsystem). The stacks of confocal 12-bit images with pixel size of 0.455 μm and a 0.684 μm Z step were acquired using a dry 20 × objective (NA 0.75). The pinhole was set to 1 AU and line average 8 was applied. Syto9 fluorescence was excited with a 488 nm laser line and 492–526 nm emission range was recorded; PI was excited with a 552 nm laser line and 565–611 nm emission range was collected. The acquisition was performed in a sequential mode. Five random fields of view per experimental condition were imaged.

Acquired images were processed and analyzed in the software Fiji/ImageJ software ver. 1.53c (NIH). First, maximum intensity projections (MIP) were obtained from stacks of images. Next, live and dead biofilm areas were established after background noise removal by thresholding and median filtering (radius 1) of respective channels. The areas from binarized images were then transferred onto original live and dead channel MIP images and mean fluorescence intensities of all detected objects per field of view were calculated using the ImageJ's Analyze Particles function.

### Statistical analysis

In this work variance analysis was performed using software Statistica 13 ver. 13.3.721.0 (ANOVA analysis). Results for which *p* < 0.05 were treated as significant.

### Ethics approval and consent to participate

This article does not contain any studies.

## Results

### Antifungal activity

*Candida albicans* ATCC 10231 was selected as an opportunistic pathogen for the study of the antifungal mechanism of action of the dicephalic surfactants investigated in this study. The sensitivity to the dicephalic surfactants—minimal inhibitory concentrations and fungicidal concentrations (MIC and MFC*) for *C. albicans* are as follows: C_14_(TAPABr)_2_ (800; > 1000* µM); C_16_(TAPABr)_2_ (400; 800* µM); C_14_(DAPACl)_2_ (80; 80* µM); C_16_(DAPACl)_2_ (16; 32* µM)^18^. Aquired results for MIC and MFC were also confirmed according to M27-A4 recommendation of the CLSI, which yelded conforming results.

### Microscopy

The results of our research on the mechanism of action of dicephalic cationic surfactants using differential interference contrast microscopy (DIC), fluorescence microscopy (FM) and transmission electron microscopy (TEM) techniques are shown below.

#### General oxidative stress

**Figure 3:**
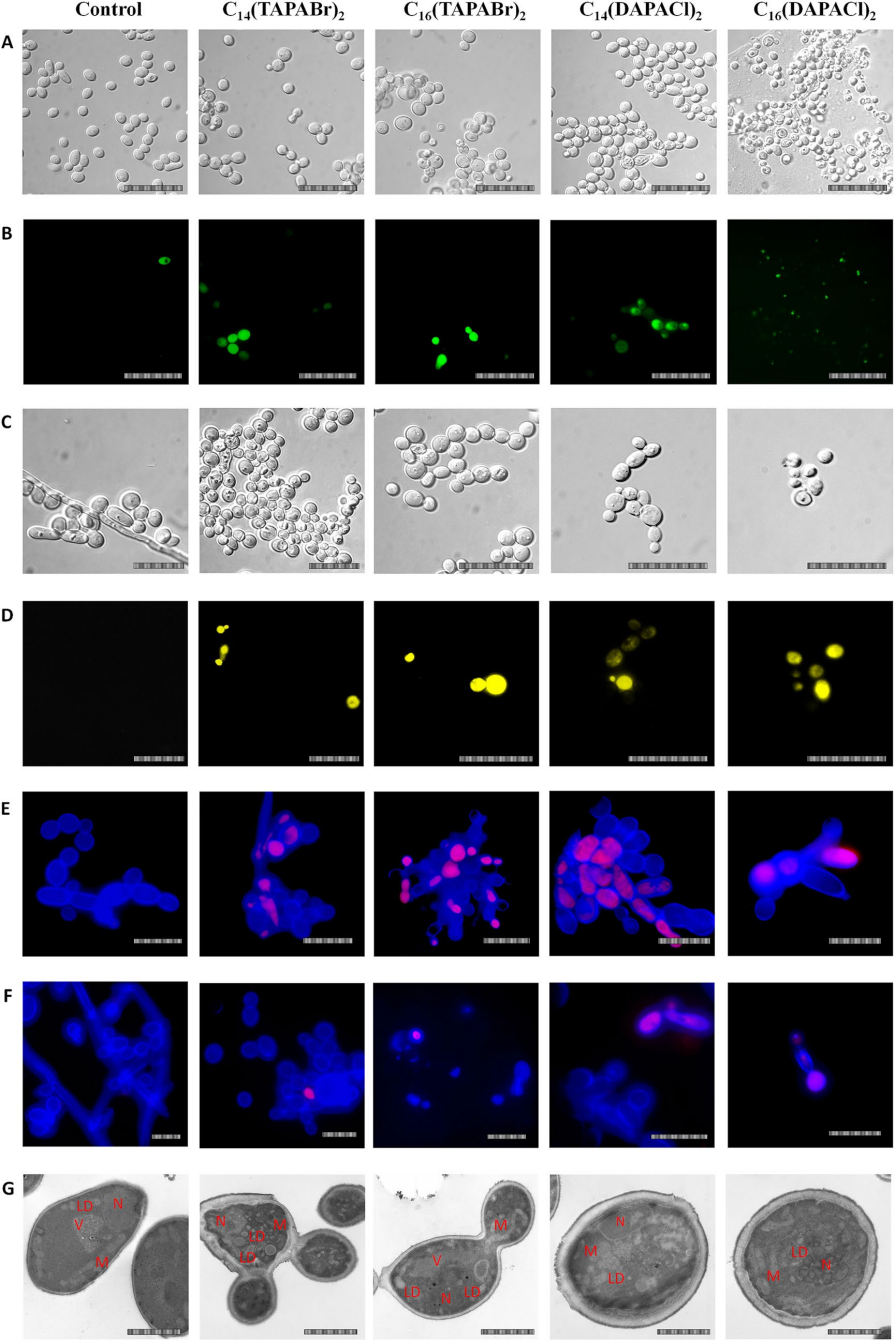
The summary illustration of the presence of intracellular oxidative stress and membrane continuity in yeast cells under the influence of the tested surfactants: general oxidative stress (green cells) (**A**,**B**), production of superoxide anion (O_2_·−) (yellow cells) (**C**,**D**) and cells with mitochondrial oxidative stress (red cells) (**E**), cells with disrupted membrane continuity show red fluorescence (**F**). DIC microscopy (**A**,**C**), fluorescence microscopy using staining: 2ʹ,7ʹ-dichlorohydrofluorescein (**B**), dihydroethidine (**D**), Calcofluor White M2R + Mitosox Red mitochondrial superoxide indicator (**E**), Calcofluor White M2R + Sytox Green Dead Cell (**F**) and TEM (**G**). Morphology of *C. albicans* cells under the influence of the studied surfactants in TEM. Notation used in the figure: *N* nucleus, *V* vacuoles, *LD* lipid droplets, *M* mitochondrion, *ER* endoplasmic reticulum; scale bars (**A**–**F**) = 10 μm and (**G**) = 500 nm.

All of the researched multifunctional cationic surfactants caused oxidative stress in C*. albicans* cells. In the untreated control about 2% of cells were phosphorescent. Selected quaternary ammonium salt (QAS) derivatives caused an increase in oxidative stress. A compound with a 14-carbon long hydrophobic chain caused an increase of oxidative stress by 32% compared to the control, while the surfactant with a 16-carbon alkyl chain caused an increase of oxidative stress by 18%. Dimethylamine derivates caused an increase in phosphorescence of cells to 40% in the case of C_14_(DAPACl)_2_, while C_16_(DAPACl)_2_ increased it to 36%. Fluorescence microscopy allowed us to confirm the induction of oxidative stress in *C. albicans* cells treated with investigated surfactants (Fig. 3A,B).

#### Production of superoxide anion

Use of fluorescence microscopy allowed us to observe an impact of dicephalic cationic surfactants on the production of the intracellular anion radical superoxide in *C. albicans.* It was found that QAS derivatives caused an increase in cells showing phosphorescence to 8% C_14_(TAPABr)_2_ and to 9% (C_16_(TAPABr)_2_. Surfactants which were diethylamine derivates caused a more significant accumulation of anion radical superoxide. C_14_(DAPACl)_2_ caused growth in the proportion phosphorescing cells to 63%, while C_16_(DAPACl)_2_ increased it to 83%. These results prove that the impact of diethylamine derivates on synthesis of anion radical superoxide is significantly stronger than those of QAS derivates (Fig. 3C,D).

#### Mitochondrial oxidative stress

Our research has shown that the surfactants we investigated could cause mitochondrial oxidative stress of *C. albicans* cells. QAS derivatives caused an increase in oxidative stress: C_14_(TAPABr)_2_ increased by 32% and C_16_(TAPABr)_2_ by 21% the number of phosphorescing cells. Surfactants derived from dimethylamine caused significantly stronger mitochondrial oxidative stress. In the presence of C_14_(DAPACl)_2_ the number of phosphorescing cells increased by 48%, in the case of C_16_(DAPACl)_2_ by 39%. Untreated cells showed no increase in phosphorescence. Diethylamine derivates induced mitochondrial oxidative stress to a further degree than QAS derivates (Fig. 3E).

#### Interruption of the cells membrane

None of the tested surfactants showed a significant ability to disrupt continuity of the *C. albicans* cell wall. QAS derivates caused quite an insignificant increase in the number of phosphorescing cells: C_14_(TAPABr)_2_ by 2% and C_16_(TAPABr)_2_ by 5%. However, C_n_(DAPACl)_2_ surfactants caused an increase in the number of cells with disrupted cell wall continuity. C_14_(DAPACl)_2_ caused an increase in the number of phosphorescing cells by 21%, while C_16_(DAPACl)_2_ caused an increase to 29% of *C. albicans* cells with disrupted membranes. No phosphorescence of control cells was observed (Fig. 3F).

#### Morphology of *C. albicans* in TEM

Transmission electron microscopy showed that the investigated surfactants could cause changes in *C. albicans* cells. In control conditions the yeast cells had shown normal morphology: vacuole, nucleus, numerous mitochondria and singular lipid droplets. Yeast cells incubated with researched QAS derivates with a 14-carbon alkyl chain had shown significant thickening of the cell wall, numerous lipid droplets in the cytoplasm and a highly granular nucleus (Fig. 3G). The 16-carbon alkyl chain surfactant had a less pronounced impact on cell morphology of yeast cells; however, numerous lipid aggregates in the cytoplasm and a slight thickening of the cell wall were noticeable. Cationic multifunctional surfactants derived from diethylamine had a stronger impact than QAS derivates. C_14_(DAPACl)_2_ and C_16_(DAPACl)_2_ caused pronounced changes in cell morphology in treated yeast cells. Cell walls were disproportionally thickened; there were numerous lipid droplets in the cells and acute morphological changes made identification of some of the organelles impossible. Dimethylamine-derived surfactants with a longer, 16-carbon alkyl chain showed a more pronounced effect on the cells than those with shorter, 14-carbon alkyl chains (Fig. 3G).

### Leakage of potassium and calcium ions

**Figure 4:**
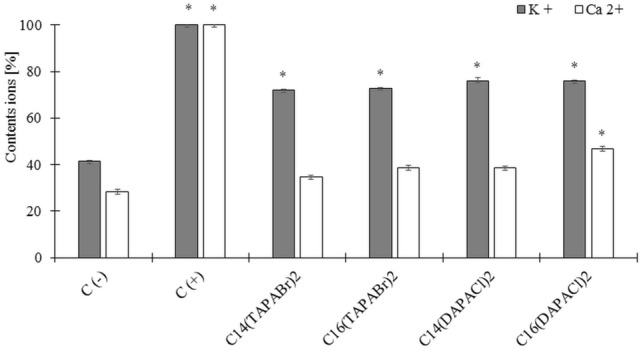
Potassium ion (K^+^) and calcium ion (Ca^2+^) leakage from *C. albicans* cells after exposure to dicephalic surfactants; C (−) surfactant untreated cells; C (+) autoclaved cells; ± SD; n = 3; *P < 0.05.

All of the analyzed surfactants significantly increased the permeability of the *C. albicans* cell membrane to potassium ions, but only C_16_(DAPA)Cl_2_ caused a small yet significant increase of permeability to calcium ions (*P* < 0.05). Permeability of the cell membrane to potassium ions was increased by about 40% by the investigated surfactants when compared to the negative control of untreated cells. In our research we did not observe a significant difference in effect, either between the groups of compounds or between compounds with different length alkyl chains, on cell membrane permeability (*P* > 0.05) (Fig. 4).

### Biofilm eradication

**Figure 5:**
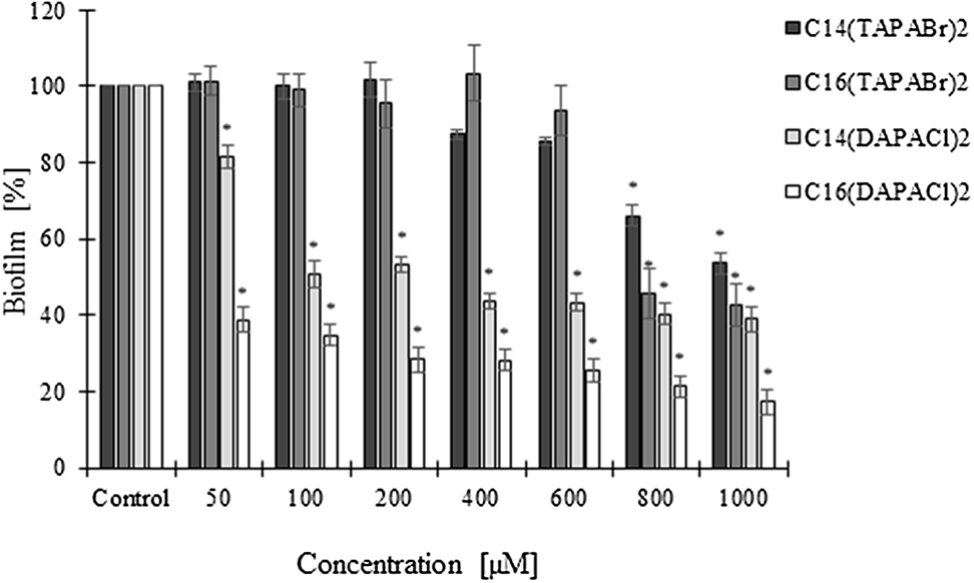
Impact of cationic dicephalic surfactants on *C. albicans* biofilm eradication on a glass surface; mean ± SD, n = 3; *significantly different from the control *P* < 0.05.

The most effective *C. albicans* biofilm eradication on glass surface for the concentration range 100–1000 μM was observed for dimethylamine derivatives (*P* < 0.05). Comparison of antibiofilm properties of cationic dicephalic surfactants shows a significant impact of the group of dimethylamine derivatives (*P* < 0.05). The length of the alkyl chain of a given derivative seemed to be of significance for biofilm eradication properties, as the strongest properties were exhibited by the derivate with the longest chain, 16 carbons long (*P* < 0.05) (Fig. 5).

### Cell viability in biofilm (CLSM)

**Figure 6:**
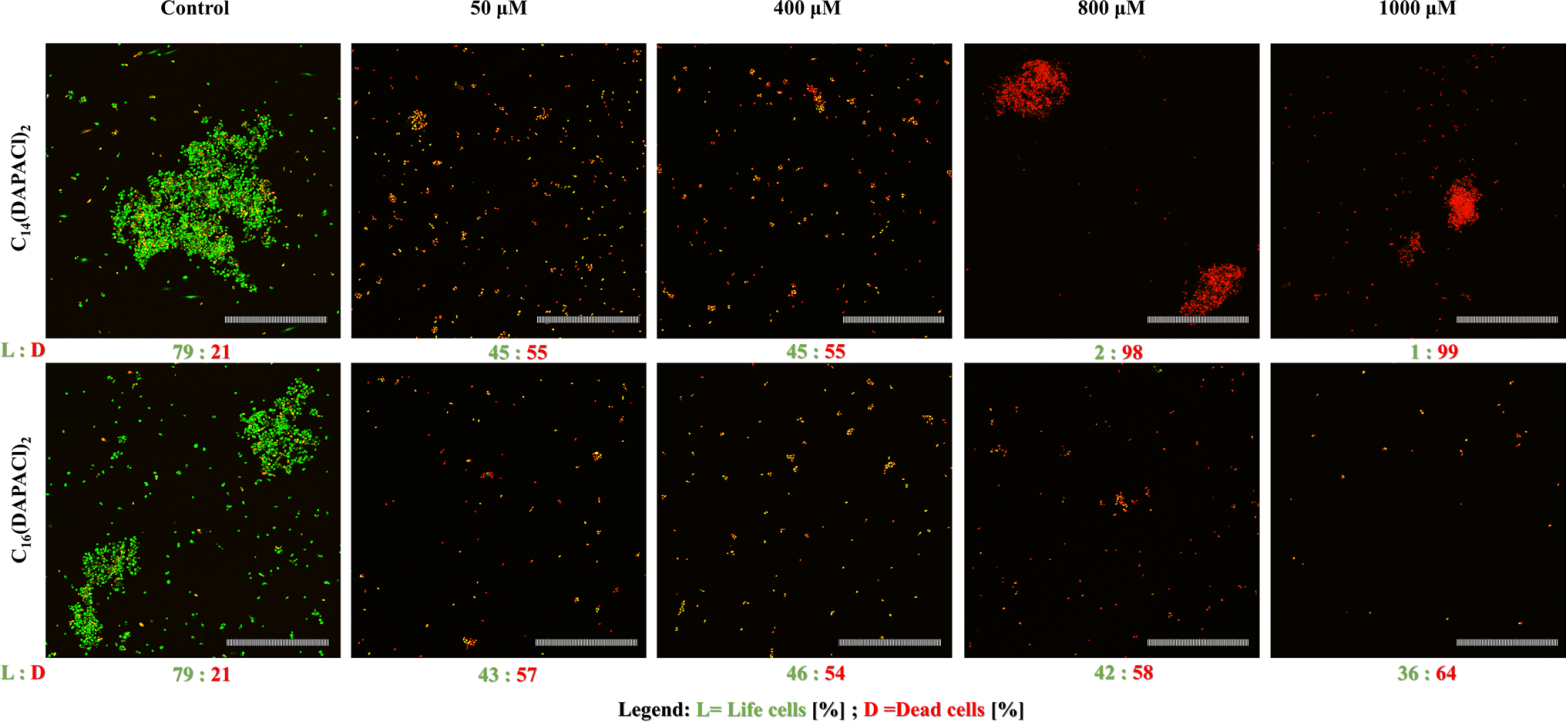
The impact of dimethylamine derived dicephalic cationic surfactants on biofilm viability of *C. albicans* on glass surface. Confocal laser scanning microscopy (CLSM): live cells (green fluorescence; Syto 9 staining) and dead cells (red fluorescence; PI staining), scale bar = 100 μm.

Confocal microscopy made it possible to observe strong antibiofilm properties of the investigated surfactants at a low concentration (50 μM) of dimethylamine derivates compared to the control on the glass surface. In the control young biofilm with almost exlusively living cells is visible. After treatment with dimethylamine derivates very significant eradication occurs, with many dead, yet still adhering to the surface, *C. albicans* cells visible, especially clearly at a concentration of 800 μM. C_14_(DAPACl)_2_ caused a strong decrease in the viability of *C. albicans* cells, ranging from 45% (50 μM) to 1% (1000 μM) viable cells. Also surfactant C_16_(DAPACl)_2_ induced a similar yet less significant decrease of viability in biofilm, reducing, *C. albicans* cell viability from 43% (50 μM) to 36% (1000 μM). Analysis of the surface of the biofilm has shown that the mode of eradication of biofilm depends on the length of alkyl chain of the surfactant. Compounds with C14 long alkyl chain induced stronger fungicidal effect, while those with C16 long chain eradicated already established biofilm more efficiently (Figs. 5, 6).

## Discussion

The broad antibacterial and antifungal properties of dicephalic cationic surfactants allow their potential application in many fields. Due to their amphiphilic properties conferred by a double hydrophilic head and singular n-variable hydrophobic alkyl tail, these compounds demonstrate the ability to adsorb on many surfaces and to cover them with a layer. This in turn may cause a reduction in the ability of microorganisms to adhere to such surfaces^18,19,29^. Compounds exhibiting the abovementioned properties are especially valuable in the light of reported resistance of *Candida* biofilms to many fungicides, including those that show an inhibitory effect on planktonic forms^30^.

This could significantly impact the complex deposition of surfactants on a surface and adhesion of yeast cells to said surface as well, including subsequent filamentation and biofilm formation^31,32^, as it has been established that cell surface hydrophobicity plays an important part in biofilm formation, so it stands to reason that changing the hydrophobicity of a surface may disrupt the process of biofilm formation or even induce its eradication^17^.

Previous research carried on cationic multifunctional surfactants seems to suggest that they may penetrate into the cell and cause disruptions in the cell metabolism, including induction of production of superoxide anion and resulting oxidative stress. Observed stronger induction of oxidative stress by 14-carbon derivates than 16-carbon ones could be best explained by the fact that surfactants which are less spatially significant may penetrate into the cells with greater ease^18^. Increased production of reactive oxygen species in cells of microorganisms results in a cytotoxic effect, often leading to apoptosis. Similar results were obtained in previous research, where antimicrobial peptides caused oxidative stress, which in turn lead to cell membrane disruption^33^. Oxidation of cell membrane lipids and sterols could also lead to increased permeability^34^.

Mitochondrial oxidative stress observed in our experiments was probably the effect of disruption of mitochondrial membranes and malfunctions of yeast electron transport chain. Increased phosphorescence results from accumulation of reactive oxygen species in endoplasmic reticulum, produced and released by mitochondria damaged by surfactants^35^ or possibly by decrease in activity of mitochondrial cytochrome c oxidase (COX)^36^. It seems that high intracellular granularity that we observed correlates with severe oxidative stress, suggesting that induction of that stress, resulting in DNA damage and finally apoptosis, may be the main mechanism of action of cationic surfactants in general, especially so that none of the surfactants that we investigated was able to cause a significant induction of cell membrane perforation. Other papers have documented those cationic surfactants such as benzalkonium chloride (BAC) could cause a genotoxic effect through induction of single and double strand DNA breakage^37^.

This is further demonstrated by our observations of potassium and calcium ions from surfactant treated cells. All investigated surfactants caused increased permeability of the *C. albicans* cell membrane to potassium ions, albeit only C_16_(DAPA)Cl_2_ had such effect regarding calcium ions. This may be rather due to induction of changes in permeability, possibly through formation of pores in the membrane, rather than its complete disintegration, as none of the surfactants caused a permeability increase comparable with that induced by positive control. Similar effect was observed in previous research, where monomeric quaternary ammonium salts were able to destabilize the cell membrane without causing its perforation^38,39^. Increase in permeability may also be an indirect effect of surfactant penetrating into a cell, causing oxidative stress and inducing oxidation of lipids and sterols by the released ROS^34,40^.

**Figure 7:**
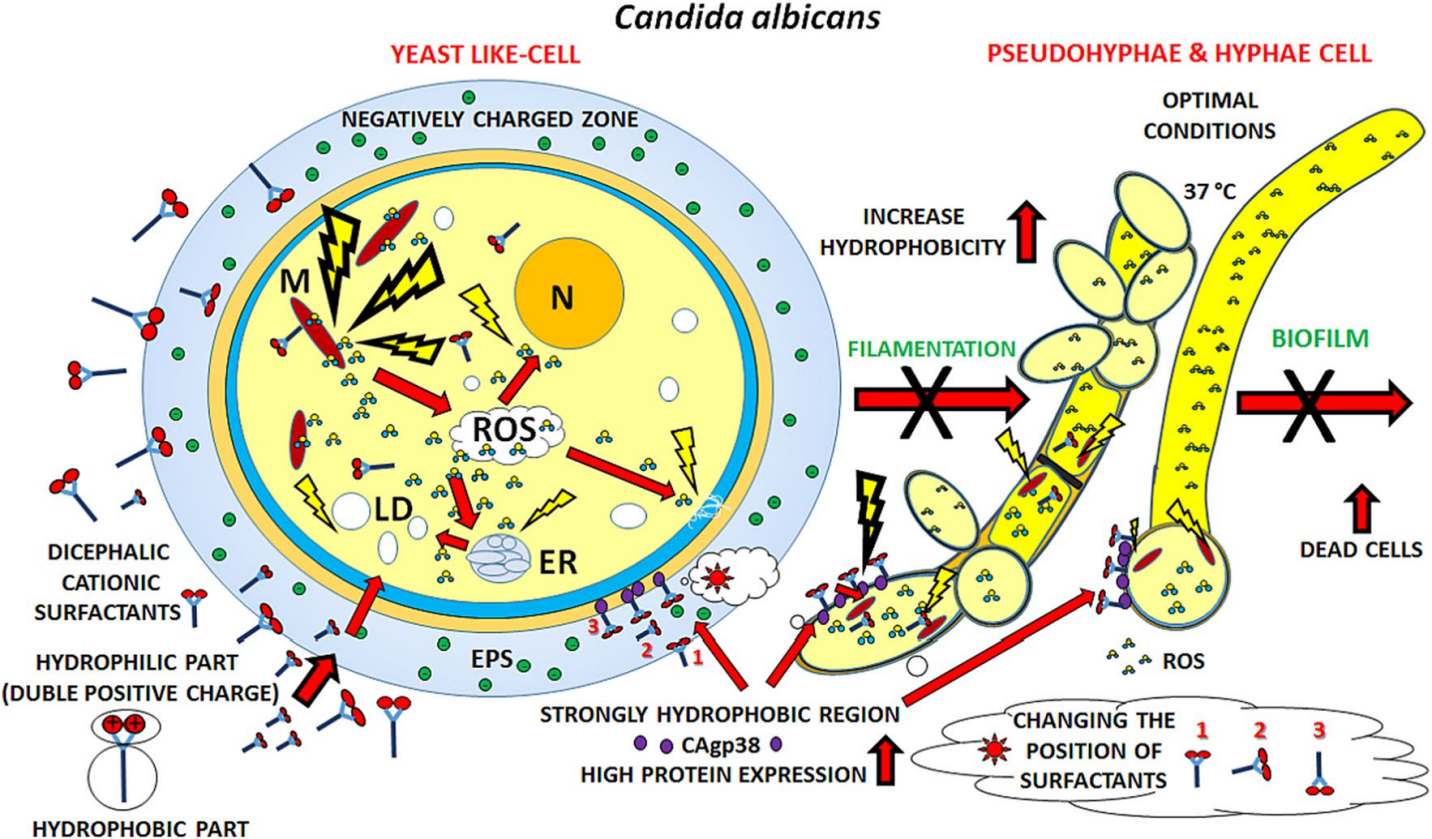
Molecular mechanism of action of dicephalic cationic surfactants on *C. albicans* cells, which takes into consideration domain structure of yeast cells, morphological changes (filamentation) of yeast-like cells to pseudohyphal and hyphal cells as well as ionic and hydrophobic interactions. Yeast cell at the first stage of adhesion starts to synthesize increased amounts of EPS (including exopolysaccharides), which determine the increase in negative surface charge of the cell, together with other cell structures. It results in the ability of double-headed cationic surfactants with their double positive charges to bind to negatively charged cell structures, orienting their hydrophilic heads towards the cell surface. Then the surfactants could interact with other areas of the yeast cell, including more hydrophobic ones, due to increased expression of proteins, which allows the surfactants to penetrate into the cell, change orientation and interact with hydrophobic molecules and cell membranes with their n-variable alkyl chains. Penetration of surfactants into the cell may cause impairment of the mitochondrial process, marked “M” in the picture, and severe oxidative stress through high production of reactive oxygen species (ROS), which in turn could cause further intracellular damage, including damage to the endoplasmic reticulum (ER) and increased synthesis of lipid droplets (LD) as well as damage to cell membranes. The multimodal mechanism of action of dicephalic surfactants may lead to a wide spectrum of intracellular damage in yeast cells, which in turn could lead to disruption of the filamentation process and biofilm formation, or lead to its significant eradication.

These processes may in turn negatively impact the cell. Negative metabolic changes caused by penetration of surfactants into the cell, as disruptions leading to morphological changes, are shown by TEM electron microscopy: cell wall thickening, cell membrane deformations, and lipid droplets in cytoplasm or oxidative stress. The mechanism of action of the researched compounds may also be dependent on the length of the alkyl chain and the precise type of hydrophilic head of a given compound, as was previously observed for surfactants^18,41^. Among the investigated surfactants a correlation between antiadhesive and antibiofilm properties on one side and the length of the alkyl chain was observed, with the surfactants with a longer chain showing greater activity. Significantly higher activity was also demonstrated by surfactants whose hydrophilic heads were derivatives of diethylamine. It is worth noting however that the activity of researched compounds depends on the material the surface they adhere to is made of^29,42^. The observed increase in activity of the compounds concurrent with increase in the length of their alkyl chains may be a result of increasing hydrophobicity of the compounds, which in turn may lead to a stronger interaction with phospholipids of the cell membranes and subsequently a disruption of their functions. Alkyl chains of the surfactants being integrated between the phospholipids of the plasma membrane could destabilize it, impairing its functions, and due to the increase in hydrophobicity lead to alkylation of highly hydrophobic surface proteins through increased affinity to them^38,43^. Under favorable conditions *C. albicans* cells adhering to a surface may modify their mosaic of surface structures during the filamentation process, among others. It impacts the hydrophobicity of cell surfaces significantly, as these structures are synthesized during filamentation. This in turn greatly impacts the adhesion process and virulence of *Candida* cells (Fig. 7)^44^.

Shorter alkyl chain cationic surfactants are generally characterized by weaker surface interactions and consequently exhibit weaker antifungal activity, showing a certain gradation, which confirms the relation between alkyl chain length and activity observed above, which is in agreement with previous research^7^. It is worth noting however that smaller particles may penetrate into the biofilm more easily and into the cells as well, for example through transmembrane water channels^45^. Cationic surfactants possessing a shorter chain are less lipophilic, so they have less of a predisposition to interact with cell membranes. Retardation of morphogenesis in *C. albicans* could be connected to disruption of polarized growth and damage of mitochondria, which seems to be confirmed by previous research in which strong oxidative stress in cells treated by surfactants was detected^46^. On a polystyrene surface a correlation between retardation of filamentation and adhesion processes and reduction of the amount of formed biofilm was observed, which was later confirmed on a glass surface^18^.

Research carried out to date suggests a positive correlation between the ability to eradicate biofilm and the length of the alkyl chain. High concentrations of surfactants could have a fungicidal effect and lead to disintegration of biofilm structure, which in the case of concentrations exceeding CMC (critical micelization concentration) could be assisted by micelization^20^. Further research carried out in this study concerning the impact of multifunctional cationic surfactants on vitality of the cells has shown its significant decrease after treatment with surfactants, especially dimethylamine derivates. It is worth noting, that our confocal microscopy observations have established a correlation not only between the effectiveness of biofilm eradication and concentration of tested surfactants, but also between said effectiveness and the length of the alkyl chain of the dimethylamine derivates. Here the positive correlation between the length of alkyl chain and ability to eradicate biofilm mentioned in the literature was confirmed for our compounds^18^. A general observation was also made, that compounds with C14 long alkyl chain induced stronger fungicidal effect, while those with C16 long chain eradicated already established biofilm more efficiently, regardless of the nature of their respective cationic heads.

## Conclusions

The research presented here enabled us to understand the biological activity of de novo synthetized cationic multifunctional surfactants, derivates of tertiary ammonia salts and dimethylamine. The investigated compounds varied in the length of their alkyl chain, which made it possible to correlate their chemical structure and mechanism of action, taking into account various models of surfaces with attention paid especially to the glass surface. Dimethylamine derived surfactants investigated in this study may be used as effective surface covering agents, limiting the ability of *C. albicans* cells to adhere to such surfaces, significantly reducing their ability to form biofilm and in higher concentrations even causing its eradication. Our research implies that the molecular mechanism of action of the investigated surfactant towards the opportunistic pathogen *C. albicans* is based on synergistic action of oxidative stress induction and disturbance of cell membranes and lipid droplet accumulation. Due to their strong antifungal activity, the surfactants which were investigated in this study could therefore be applied as effective agents eradicated biofilm (surface active agents; disinfectants).

## Supplementary Information

Supplementary Table 1.

## Data Availability

The corresponding authors will make the data available upon request.

## References

[1] Fenibo EO, Ijoma GN, Selvarajan R, Chikere CB (2019). Microbial surfactants: the next generation multifunctional biomolecules for applications in the petroleum industry and its associated environmental remediation. Microorganisms.

[2] Skrzela R, Para GY, Warszyński P, Wilk KA (2010). Experimental and theoretical approach to nonequivalent adsorption of novel dicephalic ammonium surfactants at the air/solution interface. J. Phys. Chem. B.

[3] Paluch E, Rewak-Soroczyńska J, Jędrusik J, Mazurkiewicz E, Jermakow K (2020). Prevention of biofilm formation by quorum quenching. Appl. Microbiol. Biotechnol..

[4] Bazylińska U, Zieliński W, Kulbacka J, Samoć M, Wilk KA (2016). New diamidequat-type surfactants in fabrication of long-sustained theranostic nanocapsules: Colloidal stability, drug delivery and bioimaging. Colloids Surf. B. Biointerfaces.

[5] Pérez N, Pérez L, Infante MR, García MT (2005). Biological properties of arginine-based glycerolipidic cationic surfactants. Green Chem..

[6] Tehrani-Bagha AR, Oskarsson H, Van Ginkel C, Holmberg K (2007). Cationic ester-containing gemini surfactants: Chemical hydrolysis and biodegradation. J. Colloid Interface Sci..

[7] Obłąk E, Piecuch A, Krasowska A, Łuczyński J (2013). Antifungal activity of gemini quaternary ammonium salts. Microbiol. Res..

[8] Obłąk E, Gamian A, Adamski R, Ułaszewski S (2010). The physiological and morphological phenotype of a yeast mutant resistant to the quaternary ammonium salt N-(dodecyloxycarboxymethyl)-N, N, N-trimethyl ammonium chloride. Cell. Mol. Biol. Lett..

[9] Obłąk E, Piecuch A, Guz-Regner K, Dworniczek E (2014). Antibacterial activity of gemini quaternary ammonium salts. FEMS Microbiol. Lett..

[10] Obłąk E, Piecuch A, Dworniczek E, Olejniczak T (2015). The influence of biodegradable gemini surfactants, N, N’-bis (1-decyloxy-1-oxopronan-2-yl)-N, N, N’, N’-tetramethylpropane-1, 3-diammonium dibromide and N, N’-bis (1-dodecyloxy-1-oxopronan-2-yl)-N, N, N’, N’-tetramethylethane-1, 2-diammonium dibromide, on fungal biofilm and adhesion. J. Oleo Sci..

[11] Piecuch A, Obłąk E, Guz-Regner K (2016). Antibacterial activity of alanine-derived gemini quaternary ammonium compounds. J. Surfact. Deterg..

[12] Nobile CJ, Johnson AD (2015). *Candida albicans* biofilms and human disease. Annu. Rev. Microbiol..

[13] Achkar JM, Fries BC (2010). Candida infections of the genitourinary tract. Clin. Microbiol. Rev..

[14] Calderone RA, Fonzi WA (2001). Virulence factors of *Candida albicans*. Trends Microbiol..

[15] Pappas PG, Rex JH, Sobel JD, Filler SG, Dismukes WE, Walsh TJ, Edwards JE (2004). Guidelines for treatment of candidiasis. Clin. Infect. Dis..

[16] Kullberg B, Oude AL (2002). Epidemiology of opportunistic invasive mycoses. Eur. J. Med. Res..

[17] Bujdáková H, Didiášová M, Drahovská H, Černáková L (2013). Role of cell surface hydrophobicity in *Candida albicans* biofilm. Cent. Eur. J. Biol..

[18] Paluch E, Piecuch A, Obłąk E, Wilk K (2018). Antifungal activity of newly synthesized chemodegradable dicephalic-type cationic surfactants. Colloids Surf. B. Biointerfaces.

[19] Piętka-Ottlik M, Frąckowiak R, Maliszewska I, Kołwzan B, Wilk KA (2012). Ecotoxicity and biodegradability of antielectrostatic dicephalic cationic surfactants. Chemosphere.

[20] Almeida JA, Faneca H, Carvalho RA, Marques EF, Pais AA (2011). Dicationic alkylammonium bromide gemini surfactants. Membrane perturbation and skin irritation. PLoS ONE.

[21] Okińczyc P, Paluch E, Franiczek R, Widelski J, Wojtanowski KK, Mroczek T, Krzyżanowska B, Skalicka-Woźniak K, Sroka Z (2020). Antimicrobial activity of *Apis mellifera* L. and *Trigona* sp. propolis from Nepal and its phytochemical analysis. Biomed. Pharmacother..

[22] Kostakioti M, Hadjifrangiskou M, Hultgren S (2013). Bacterial biofilms: development, dispersal, and therapeutic strategies in the dawn of the postantibiotic era. Cold Spring Harb. Perspect. Med..

[23] Cammarota G, Sanguinetti M, Gallo A, Posteraro B (2012). Review article: biofilm formation by *Helicobacter pylori* as a target for eradication of resistant infection. Aliment. Pharmacol. Ther..

[24] Wawrzyńczyk D, Bazylińska U, Lamch Ł, Kulbacka J, Szewczyk A, Bednarkiewicz A, Wilk KA, Samoć M (2019). Förster resonance energy transfer-activated processes in smart nanotheranostics fabricated in a sustainable manner. Chemsuschem.

[25] Begec Z, Yucel A, Yakupogulları Y, Erdogan MA, Duman Y, Durmus M, Ersoy MO (2013). The antimicrobial effects of ketamine combined with propofol: An in vitro study. Rev. Bras. Anestesiol..

[26] Clinical and Laboratory Standards Institute (2017) Reference method for broth dilution antifungal susceptibility testing of yeasts. Approved standard-fourth edition. CLSI document M27- A4.Wayne, PA

[27] Reynolds ES (1963). The use of lead citrate at high pH as an electron-opaque stain in electron microscopy. J. Cell Biol..

[28] Obłąk E, Piecuch A, Maciaszczyk-Dziubińska E, Wawrzycka D (2016). Quaternary ammonium salt N-(dodecyloxycarboxymethyl)-N, N, N-trimethyl ammonium chloride induced alterations in *Saccharomyces cerevisiae* physiology. J. Biosci. (Bangalore).

[29] Piecuch A, Lamch Ł, Paluch E, Obłąk E, Wilk K (2016). Biofilm prevention by dicephalic cationic surfactants and their interactions with DNA. J. Appl. Microbiol..

[30] Chandra J, Mukherjee PK (2015). *Candida* biofilms: Development, architecture, and resistance. Microbiol. Spectrum.

[31] Paria S, Khilar KC (2004). A review on experimental studies of surfactant adsorption at the hydrophilic solid–water interface. Adv. Colloid Interface Sci..

[32] Cavalheiro M, Teixeira MC (2018). *Candida* biofilms: Threats, challenges, and promising strategies. Front. Med..

[33] Wang K, Dang W, Xie J, Zhu R, Sun M, Jia F, Zhao Y, An X, Qiu S, Li X, Ma Z, Yan W, Wang R (2015). Antimicrobial peptide protonectin disturbs the membrane integrity and induces ROS production in yeast cells. Biochim. Biophys. Acta.

[34] Böcking T, Barrow KD, Netting AG, Chilcott TC, Coster HG, Höfer M (2000). Effects of singlet oxygen on membrane sterols in the yeast *Saccharomyces cerevisiae*. Eur. J. Biochem..

[35] Murphy MP (2013). Mitochondrial dysfunction indirectly elevates ROS production by the endoplasmic reticulum. Cell Metab..

[36] Leadsham JE, Sanders G, Giannaki S, Bastow EL, Hutton R, Naeimi WR, Breitenbach M, Gourlay CW (2013). Loss of cytochrome c oxidase promotes RAS-dependent ROS production from the ER resident NADPH oxidase, Yno1p, in yeast. Cell Metab..

[37] Ye J, Wu H, Zhang H, Wu Y, Yang J, Jin X, Shi X (2011). Role of benzalkonium chloride in DNA strand breaks in human corneal epithelial cells. Graefes Arch. Clin. Exp. Ophthalmol..

[38] Ioannou CJ, Hanlon GW, Denyer SP (2007). Action of disinfectant quaternary ammonium compounds against *Staphylococcus aureus*. Antimicrob. Agents Chemother..

[39] Shirai A, Sumitomo T, Kurimoto M, Maseda H, Kourai H (2009). The mode of the antifungal activity of gemini-pyridinium salt against yeast. Biocontrol Sci..

[40] Dawes IW, Perrone GG (2019). Stress and ageing in yeast. FEMS Yeast Res..

[41] Ferreira GF, dos Santos-Pinto BL, Souza EB, Viana JL, Zagmignan A, dos Santos JRA, Santos ÁRC, Tavares PB, Denadai ÂML, Monteiro AS (2016). Biophysical effects of a polymeric biosurfactant in *Candida krusei* and *Candida albicans* cells. Mycopathologia.

[42] Liu WS, Wang CH, Sun JF, Hou GG, Wang YP, Qu RJ (2015). Synthesis, characterization and antibacterial properties of dihydroxy quaternary ammonium salts with long chain alkyl bromides. Chem. Biol. Drug Des..

[43] Deo A, Kulkarni A, Meshram S (2010). Monocationic surfactant induced ultra structural changes in antibiotic resistant *Escherichia coli*. Indian J. Med. Res..

[44] Hazen KC, Wu JG, Masuoka J (2001). Comparison of the hydrophobic properties of *Candida albicans* and *Candida dubliniensis*. Infect. Immun..

[45] Kuhn D, Chandra J, Mukherjee P, Ghannoum M (2002). Comparison of biofilms formed by *Candida albicans* and *Candida parapsilosis* on bioprosthetic surfaces. Infect. Immun..

[46] Yu Q, Zhang B, Ma F, Jia C, Xiao C, Zhang B, Xing L, Li M (2015). Novel mechanisms of surfactants against *Candida albicans* growth and morphogenesis. Chem. Biol. Interact..

