# Supplementary material for: Biofilm eradication and antifungal mechanism of action against Candida albicans of cationic dicephalic surfactants with a labile linker
Source: Sci Rep. 2021 Apr 26;11:8896. doi: 10.1038/s41598-021-88244-1 (PMC8076202; doi:10.1038/s41598-021-88244-1)
Supplement: Supplementary file 1 — Supplementary Table 1. [file 41598_2021_88244_MOESM1_ESM.pdf]

Scientific Reports

Biofilm eradication and antifungal mechanism of action against *Candida albicans* of cationic dicephalic surfactants with a labile linker

Emil Paluch [a*], Jakub Szperlik [b], Łukasz Lamch [c], Kazimiera A. Wilk [c], Ewa Obłąk [d*]

[a] Department of Microbiology, Faculty of Medicine, Wroclaw Medical University, Tytusa Chałubińskiego 4, 50-376 Wrocław, Poland

[b] Department of Genetic Biochemistry, Faculty of Biotechnology, University of Wroclaw, Przybyszewskiego 63, 51-148 Wroclaw, Poland

[c] Department of Engineering and Technology of Chemical Processes, Faculty of Chemistry, Wrocław University of Science and Technology, Wybrzeże Wyspiańskiego 27, 50-370 Wroclaw, Poland

[d] Department Physicochemistry of Microorganisms, Institute of Genetics and Microbiology, University of Wrocław, Przybyszewskiego 63/77, 51-148 Wrocław, Poland

[*] Corresponding author: emil.paluch@umed.wroc.pl; ewa.oblak@uwr.edu.pl

Electronic Supplementary Materials

Tables

Table 1

Chemical structures and abbreviations of dicephalic surfactants [18]

Structure	R	Abbreviation	M [g/mol]
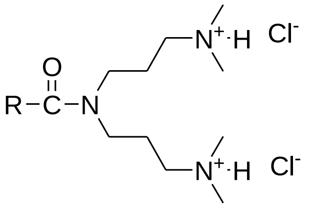	*n*-C_9H_{19}	$C_{10}(DAPACl)_2$	414.49
	n-$C_{11}H_{23}$	$C_{12}(DAPACl)_2$	442.55
	n-$C_{13}H_{27}$	$C_{14}(DAPACl)_2$	470.60
	n-$C_{15}H_{31}$	$C_{16}(DAPACl)_2$	498.66
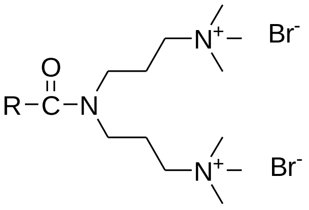	*n*-C_9H_{19}	$C_{10}(TAPABr)_2$	531,49
	n-$C_{11}H_{23}$	$C_{12}(TAPABr)_2$	559.55
	n-$C_{13}H_{27}$	$C_{14}(TAPABr)_2$	587.60
	n-$C_{15}H_{31}$	$C_{16}(TAPABr)_2$	615.66
